# Campus Nature Rx: How investing in nature interventions benefits college students

**DOI:** 10.3389/fpsyg.2022.960370

**Published:** 2022-07-25

**Authors:** Donald A. Rakow, Dorothy C. Ibes

**Affiliations:** ^1^Cornell University, Ithaca, NY, United States; ^2^College of Agriculture and Life Sciences, Cornell University, Ithaca, NY, United States; ^3^Section of Horticulture, College of Agriculture and Life Sciences, Cornell University, Ithaca, NY, United States; ^4^Environmental Science and Policy, Center for Geospatial Analysis, William & Mary, Williamsburg, VA, United States

**Keywords:** campus, green infrastructure, mental health, Nature Rx, biophilic

## Introduction

The college experience is a time of discovery, learning, and personal growth. However, many U.S. college students report unprecedentedly high levels of mental health problems during these formative years. According to the 2021 National Collegiate Health Assessment, over 30 percent of students received psychological or mental health services in the previous 12 months. These challenges impact academic performance, with student classroom effectiveness reportedly reduced 22% due to depression, and nearly 38% due to stress (American College Health Assessment, 2022). Further, psychological issues are worsening. The most recent American Freshman National Survey revealed that incoming college students' self-reported physical and emotional health has been steadily declining since 1985 (Stolzenberg et al., 2020). Anxiety, depression, and sleep disorders are especially prevalent among college students (Pedrelli et al., 2015). In a 2020 national survey, two-thirds of student respondents cited loneliness as a distinct problem, an all-time high that reflects the mental health impact of the COVID-19 pandemic, including social distancing and other measures (McAlpine, 2021). Alongside these other stressors, chronic loneliness can, in extreme cases, cause young people to consider suicide. A pediatric emergency department study found a significant increase in suicide-risk among youth aged 11–21, before, vs. after the onset of the pandemic (Hill et al., 2021). In another national study, suicide death rates were highest among American Indian and Alaska Native people, males, and residents of rural areas (Saunders and Nirmita, 2022).

There are numerous underlying causes of this mental health crisis among college-aged youth. Among these are the effects of academic and financial pressures, relationship issues, information overload, anxiety about the future, screentime, and social media (Pedrelli et al., 2015). Rising campus mental health issues have placed unsustainable demands on college health clinics, particularly counseling and psychological service (CAPS) units. One study estimated that colleges with student populations of 15,000 spend on average $750,000 annually on student mental health care (Eisenberg, 2015). Further, higher caseloads per counselor are associated with fewer sessions per student, less frequent appointments, reduced improvement in symptoms, and burnout among clinicians (American College Health Assessment, 2022).

## Nature as a preventative, non-pharmacological intervention

In recent decades, an impressive number of studies have provided scientific evidence for the mental, and other health benefits of nature engagement. Among these benefits are reductions in stress (Antonelli et al., 2019; Hunter et al., 2019), anxiety (Bratman et al., 2015), and depression (Kondo et al., 2018), as well as improved memory recall, concentration, sleep patterns, and overall mood (Berman et al., 2008; Bratman et al., 2012). A 2017 review provided a comprehensive listing of the psychological, physiological, and behavioral benefits that can be derived from time in nature, as well as further research that needs to be conducted on each of these (Frumkin et al., 2017). Given the ample positive effects of nature exposure, a movement has arisen in recent years whereby healthcare providers prescribe time outdoors, to improve their patient's physical and mental health. These programs include Park Rx, Nature Rx, Walk with a Doc, Healthy Parks Healthy People, Nature as Medicine, among others.

The importance of time in nature for mental health and wellbeing also served as the nucleus of what has evolved into the Campus Nature Rx (CNRx) Network. From an initial partnership of four schools in 2019– Cornell University, University of California- Davis, University of Minnesota, and William & Mary– the network is now a coalition of over 50 U.S. colleges and universities (for a full list of current members, visit campusnature.com).

The Campus Nature Rx approach is based on the belief that a university education involves more than academics, addressing the whole person, including their connection to the natural world. Such programs support a sense of place and belonging at these institutions and are consistent with studies that have shown college students' valuing and use of green spaces on their campuses (Speake et al., 2013). Another unifying understanding among CNRx members is that any campus can support nature engagement, outside or indoors, *via* greenspace, gardens, trees, plants, nature imagery, biophilic design, and other approaches. Further, nature exposure can accommodate even the busiest student schedules. Research has shown that 1 and 5-min green microbreaks on campus greenspaces effectively support stress-relief (Ibes et al., 2018). Nature experiences of between 10 and 20 min have been shown to improve mood an average of 86% among student participants (Ibes and Forestell, 2022), and can significantly and positively impact psychological and physiological markers for college-aged individuals (Meredith et al., 2020). Given the theme of this special issue, the time duration of 20–30 min has been found to most efficient, after which physiological benefits continued to accrue but at a reduced rate (Hunter et al., 2019).

## Campus Nature Rx approaches

At their respective institutions, members of the CNRx Network apply a diverse set of approaches to provide nature exposure and encourage nature engagement on their campuses. In many cases, members are collecting data to evaluate the reach and impact of such efforts. The primary approaches can be organized into the categories of Nature Rx programs, physical infrastructure, online maps, courses, communications, and nature-oriented activities.

### Nature Rx programs

Since, 2017 professionals at the Cornell Health clinic have prescribed time in nature to students through electronic health records. During the '21-'22 academic year, 406 students received nature prescriptions, and 36 percent responded to a follow-up survey. At University of Kentucky, an interdisciplinary team is working with healthcare leadership to design and implement a pilot Nature Rx project adjacent to cancer treatment clinics. At William & Mary, a peer referral approach is employed by the Parks and Ecotherapy Research Lab (PERL) Campus Park Rx program, established in 2014. Trained Peer Park Ambassadors use a database to refer fellow students to one of over 100 local greenspaces based on their interests, needs, transportation options, and schedule. During the academic year, over 100 students receive a referral *via* an online form or tabling event on campus.

### Green infrastructure

Some programs have focused on making existing outdoor spaces more comfortable, welcoming, and convenient. At Cornell, student-built sod sofas were placed around campus, Swarthmore provided oversized chairs on campus greens, and at William & Mary chair-bombing provides comfortable seating in underutilized campus greenspace. At University of Kentucky, the student-informed, interdisciplinary Mindful Oasis project partnered with Facilities Management to provide intentional spaces for wellness campus-wide, including pop-up seating areas. California State University Monterey Bay (CSUMB) was the first university to undertake the Living Community Challenge as part of its master planning processes. In a Research Methods class, students review the basic biophilic design elements, then use colored frames to highlight aspects of campus that feel supportive (green frames) or that they would change (red frames) to make the landscape more biophilic. In terms of mental restoration, the majority of students emphasized the importance of light and views from interiors because that is where they spend the majority of their time, both studying and working. They also expressed wanting to see more blurring between the indoors and out, with interior gardens or plantings, and windowed areas that lead out to natural courtyards.

### Online maps

Some CNRx members have developed online maps to support time outdoors. In March of 2020, William & Mary's PERL released their Campus Greenspace Map. Ten interactive maps display photos and details for 12 birding sites, 50 significant trees, and more than 100 greenspaces, hiking trails, sport areas, patios, and other outdoor spaces on campus. The sites are organized by activity (e.g., studying, relaxing, eating), so users can quickly find outdoor spaces that fit their needs and preferences, while helping them get their daily dose of nature. The map series is accessible *via* the official William & Mary app, and online (campusgreenspace.wm.edu). Members of NatureRx at UConn mapped 122 miles of hiking trails on and near campus. Mapped sites feature a description with photographs, alongside a student-produced guide to activities for engaging with nature (see naturerx.initiative.uconn.edu).

### Courses

Both Cornell and UC Davis offer Nature Rx courses specific to their respective campuses. Pre- and post-surveys in both courses over a 2 year period demonstrated that students associate participation in the course with a strengthened belief in the value of spending time in nature to reduce stress, the creation and solidification of social bonds, and an expectation that the class would have a lasting impact (Kiers et al., 2021). At William & Mary, The Science and Experience of Ecotherapy has been a course offering since 2017. Validated pre- and post-surveys found that nature connectedness increased significantly following the course, and weekly 80-min ecotherapy practicums increased multiple dimensions of mood among students an average of 56%. Campus Nature Rx courses are also an opportunity to address inequity and barriers to nature access. A course at the University of Maryland, Black Bodies and Green Spaces: From 1619 to Today, critically examines how systemic racism has shaped the experience, connection, and relationship to nature among Black Americans. It also explores how many Black Americans regard nature as a space of freedom, humanity, and spirituality. At some institutions, a Nature Rx component has been a successful addition to existing course offerings. At the University of Minnesota, a virtual forest bathing experience was added to the first 20 min of each class in an evidence-based nursing practice and research course. The students reported a reduction in perceived stress and feelings of calm and increased awareness of the importance of taking time for oneself.

### Communications and outreach

Campus Nature Rx programs utilize various modes of communication to support nature appreciation and engagement including social media, websites, newsletters, and media walls. Social media platforms including Facebook, Instagram, Twitter and TikTok are used by programs at UC Davis, U of Maryland, and W&M. William & Mary's PERL 2021 Greenspace March Madness and Where's Walnut Social Media campaigns garnered over 4,000 interactions in less than a month, and the program's website (which shares nature and health resources, research, and events), attracts an average of over 2,000 unique visitors a year. The UC Davis and W&M programs host regular newsletters, while media walls around the Cornell campus display messages of the benefits of time in nature.

### Nature-oriented activities

CNRx programs are continuously experimenting with new ways to engage students with campus nature. Leaders at UC Davis have organized a Nature Rx Campus Community Health and Wellbeing Series, Sheep mowers, Chair Share Program, Learning by Leading internship program, and Public Outreach and Engagement activities including stargazing, Yoga in the Arboretum, Arboretum Bingo, and Nature Rx photo Scavenger Hunt. NatureRx at UConn has hosted a mini-symposium and Room to Grow, a house plant workshop, and co-sponsored an event called “Forest Bathing in the HEEP Forest.” U of Maryland Master of Public Health students and faculty organized a forest bathing session. PERL at W&M has served over 200 students with programs including a Bird Scavenger Hunt and Map, Campus Tree Tours, and Greenspace Scavenger Hunt. The University of Florida produces illustrated nature guides that include images and descriptions of local organisms to increase students' awareness of and excitement about nearby nature. At U of Kentucky, CNRx members host wellness coaching, Walk with a Doc, tree walks, and tree week.

## Discussion

The COVID pandemic had some administrators questioning the need for residential college experiences, particularly given the high cost of campus maintenance. The burgeoning Campus Nature Rx movement provides compelling evidence that on-campus nature experiences provide a high return on investment by offering scientifically proven, equitable, and cost-effective solutions for improving college student mental health, among other benefits. Quantitative and qualitative data reveal that students value the natural elements and spaces on their campuses, as well as efforts to enhance their engagement with them. This sentiment was expressed by a Cornell Nature Rx student who wrote in a semester-end assessment, “Thank you so much for making my last semester at Cornell so special. Your class has been really impactful and I'm grateful for what we learned and experienced.” Likewise, in an anonymous evaluation of the Science & Experience of Ecotherapy course, a Spring 2020 William & Mary graduate wrote, “There has been no more essential class in my career at WM—essential to my personal health, empowerment for the pursuit of my values, confidence in the expression of myself in the natural world and the increasing awareness of the beauty unfolding around me at all times. I feel closer to myself and more equipped to maneuver the adversity of life moving forward as a result of this class.” In the midst of a college mental health crisis exacerbated by a global pandemic, CNRx offerings represent cost-effective and meaningful approaches for bolstering psychological resilience, helping students, and colleges, grow and thrive.

## Author contributions

This opinion piece was co-authored by DR and DI, with much input from various members of the Campus Nature Rx Network. All authors contributed to the article and approved the submitted version.

## Conflict of Interest

The authors declare that the research was conducted in the absence of any commercial or financial relationships that could be construed as a potential conflict of interest.

## Publisher's note

All claims expressed in this article are solely those of the authors and do not necessarily represent those of their affiliated organizations, or those of the publisher, the editors and the reviewers. Any product that may be evaluated in this article, or claim that may be made by its manufacturer, is not guaranteed or endorsed by the publisher.
